# Elicitation of Inhibitory Effects for AGE-Induced Oxidative Stress in Rotator Cuff-Derived Cells by Apocynin

**DOI:** 10.3390/cimb45040225

**Published:** 2023-04-14

**Authors:** Takahiro Furukawa, Takashi Kurosawa, Yutaka Mifune, Atsuyuki Inui, Hanako Nishimoto, Yasuhiro Ueda, Takeshi Kataoka, Kohei Yamaura, Shintaro Mukohara, Tomoya Yoshikawa, Issei Shinohara, Tatsuo Kato, Shuya Tanaka, Masaya Kusunose, Yuichi Hoshino, Takehiko Matsushita, Ryosuke Kuroda

**Affiliations:** Department of Orthopaedic Surgery, Kobe University Graduate School of Medicine, 7-5-2 Kusunoki-Cho, Chuo-ku, Hyogo, Kobe 650-0017, Japan

**Keywords:** advanced glycation end-products, apocynin, oxidative stress, reactive oxygen species, rotator cuff

## Abstract

Advanced glycation end-products (AGEs) play a critical supportive role during musculoskeletal disorders via glycosylation and oxidative stress. Though apocynin, identified as a potent and selective inhibitor of NADPH oxidase, has been reported to be involved in pathogen-induced reactive oxygen species (ROS), its role in age-related rotator cuff degeneration has not been well clarified. Therefore, this study aims to evaluate the in vitro effects of apocynin on human rotator cuff-derived cells. Twelve patients with rotator cuff tears (RCTs) participated in the study. Supraspinatus tendons from patients with RCTs were collected and cultured. After the preparation of RC-derived cells, they were divided into four groups (control group, control + apocynin group, AGEs group, AGEs + apocynin group), and gene marker expression, cell viability, and intracellular ROS production were evaluated. The gene expression of NOX, IL-6, and the receptor for AGEs (RAGE) was significantly decreased by apocynin. We also examined the effect of apocynin in vitro. The results showed that ROS induction and increasing apoptotic cells after treatment of AGEs were significantly decreased, and cell viability increased considerably. These results suggest that apocynin can effectively reduce AGE-induced oxidative stress by inhibiting NOX activation. Thus, apocynin is a potential prodrug in preventing degenerative changes of the rotor cuff.

## 1. Introduction

Rotator cuff tears are one of the most common disorders associated with pain and dysfunction in the shoulder. Yamamoto et al. reported that 20.7% of 1366 shoulders, with a mean age of 57.9 years, had full-thickness rotator cuff tears [1]. Most rotator cuff tears result from age-related degenerative changes. Several etiologies have been related to the pathophysiology of rotator cuff tears, including intrinsic variables such as age, diabetes, and hypercholesterolemia [2] and extrinsic variables such as subacromial and internal impingement, tensile overload, and repetitive minor injury [3]. Rotator cuff degeneration has been characterized by pathologic changes such as thinning and disorientation of collagen fibers, as well as loss of cellularity, vascularity, and fibrocartilage mass at the site of cuff insertion [3].

Recently, advanced glycation end-products (AGEs) and advanced lipoxidation end-products (ALEs) have been gaining attention because the deposition of AGEs and ALEs in organs and tissues can cause various diseases, such as arteriosclerosis [4], renal failure [5], and osteoporosis [6,7,8]. Due to covalent cross-linking, the development of AGEs and ALEs impairs the structure of proteins, causing oligomerization and aggregation. Cellular function changes, cell damage, and death result from this. For instance, several studies have found that the mitochondrial, endoplasmic reticulum, and extracellular matrix (ECM) proteins are impaired, as well as those involved in the cell cycle and gene expression regulation [9,10]. Furthermore, oxidative stress and the AGEs and ALEs that result from it have been linked to aging and many chronic age-related disorders [11,12]. Attaching to the AGEs receptor, AGEs cause oxidative stress and inflammation to rise (RAGE) [13]. Many AGEs accumulate in collagen, which might result in aberrant covalent intermolecular connections and interfere with interactions between cells and the matrix [14]. The link between AGEs and reactive oxygen species (ROS) has recently been established, and ROS are essential for senescence [15]. ROS, the significant oxidative stress component, is responsible for cellular and tissue damage. ROS can damage DNA, RNA, and proteins and alter the amounts of antioxidant enzymes [16]. According to earlier research, nicotinamide adenine dinucleotide phosphate oxidase (NOX) is the primary ROS source, and its activation enhances ROS generation [17]. A crucial role in starting and accelerating the development of diabetic complications is played by ROS produced from NOX [18]. According to Mifune et al., both in vitro and ex vivo experiments revealed that AGEs had negative effects on rotator cuff-derived cells, including reduced cell viability, increased ROS production, cell death, and lower tensile strength [19]. Therefore, reducing AGEs’ ability to cause oxidative stress might help to prevent aging-related deterioration of the rotator cuff [19].

The NOX inhibitor apocynin, isolated from the plant Picrorhiza kurroa [20], has been shown to substantially reduce intracellular ROS [20]. It has been extensively studied as an antioxidant agent in experimental research. However, most findings have been reported in endothelium and vascular smooth muscle cells [20,21]. Furthermore, chronic apocynin treatment dramatically reduced restricted skeletal muscle mobility and mitochondrial dysfunction in mice given a high-fat diet [22,23]. As far as we know, the effects of apocynin on cells obtained from human rotator cuffs have not been studied. Therefore, we hypothesize that apocynin will exert antioxidant effects on AGE-induced oxidative stress in human-derived cells. Therefore, this study aimed to evaluate in vitro apocynin effects on human rotator cuff-derived cells.

## 2. Materials and Methods

### 2.1. Ethics Statement

All experiments were conducted under the approval and guidance of the ethics committee of our institute, and informed consent was obtained from all patients.

### 2.2. Preparation and Treatment of Human Rotator Cuff-Derived Cells

The institutional review board approved the protocol of this study. During arthroscopic rotator cuff repair, human rotator cuff tissues were extracted from the damaged margins of supraspinatus tendons. Twelve patients with rotator cuff tears were enrolled, with an average age of 64.8 ± 11.7 SD. Patient background characteristics are summarized in Table 1. The human adult rotator cuff tissues were transported in a sterile saline solution on ice. Tissues were cut into small (12 mm) pieces and plated onto dishes with Dulbecco’s Modified Eagle’s Medium (Sigma, St. Louis, MO, USA) supplemented with 10% fetal bovine serum and 1% penicillin–streptomycin (Sigma, St. Louis, MO, USA) (regular medium) under sterile conditions to isolate rotator cuff-derived cells. The dishes were maintained at 37 °C with 95% humidity and 5% CO_2_. After adhesion of cells to dishes was observed, the cells were washed in phosphate-buffered saline (PBS), detached using 0.05% trypsin−0.02% EDTA (WAKO, Osaka, Japan), and pelleted in 75 cm^2^ cell culture flasks with a regular medium. The cultures obtained from each patient were gathered into one for each examination. The cells were seeded onto 12-well plates at 5.0 × 10^4^ cells per well. After the cells had reached 70% confluence, they were cultured in two groups: regular medium only (control group) and regular medium supplemented with 100-µg/mL AGEs BSA (Trans Genic, Kobe, Japan) (AGEs group). A 2 mM stock solution of apocynin (Tokyo Chemical Industry, Tokyo, Japan) was dissolved in dimethyl sulfoxide (DMSO) and diluted to a final concentration of 100 μM. For cell seeding, 100 μM apocynin was added to each group. The cells were ultimately separated into four groups: (1) the control group, (2) the control group with apocynin (control + apo group), (3) the AGEs group, and (4) the AGEs group with apocynin (AGEs + apo group). All experiments were conducted with cells from passages 2 to 3, and the same passage of cells was used for each experiment.

### 2.3. Quantitative Real-Time Polymerase Chain Reaction (PCR)

At 48 h, total RNA was extracted from tenocytes using an RNeasy Mini kit (Qiagen, Valencia, CA, USA). We reverse-transcribed total RNA into single-strand cDNA using a High-Capacity Reverse Transcription Kit (Applied Biosystems, Foster City, CA, USA). Using an Applied Biosystems 7900 HT Fast Real-Time PCR System and SYBR Green reagents, triplicate real-time PCR was performed on the cDNA (Applied Biosystems). Results were normalized to housekeeping gene expression levels and expressed relative to the control (untreated) culture levels using the 2^−ΔΔCt^ method. The primers for nicotinamide adenine dinucleotide phosphate (NOX)-1, NOX-4, interleukin-6 (IL-6), and the receptor for AGEs (RAGE) were then used. The primer sequences are listed in Table 2.

### 2.4. Cell Viability Assay

We assessed cell proliferation with Cell Counting Kit-8 and a water-soluble tetrazolium salt (WST) assay (Dojindo, Kumamoto, Japan). Briefly, we utilized 96-well plates and seeded each well with 2000 cells and 100 μL of the medium. Before the WST test analysis, each 96-well plate was grown at 5% CO_2_ and 37 °C. We then exposed the cells for 48 h to four different mediums (control, control + apo, AGEs, and AGEs + apo). We supplemented each well with 10 μL WST for 4 h at 5% CO_2_ and 37 °C before evaluation for the WST assay. At 450 nm, we assessed the conversion of WST to formazan spectrophotometrically.

### 2.5. Apoptotic Cells Analysis

We identified nuclear fragmentation by TdT-mediated dUTP Nick End Labeling (TUNEL) with an APO-DIRECTTM KIT (PHOENIX FLOW SYSTEMS, San Diego, CA, USA) according to the manufacturer’s instructions, utilizing fixed cells (4% paraformaldehyde/phosphate-buffered saline) with 2-(4-aminophenyl)-1H-indole-6-carboxamidine (DAPI). For quantitative analysis, we counted apoptosis-positive and DAPI-positive cells in four rectangular areas (0.75 mm 1.0 mm) on each slide and calculated the mean results. The number of apoptosis-positive nuclei divided by the number of DAPI-positive nuclei was multiplied by 100 to obtain the percentage of apoptosis-positive cells, which was then represented as the average across the four locations.

### 2.6. Detection of ROS Production

According to the manufacturer’s instructions, we used the Total ROS/Superoxide Detection Kit (Enzo Life Science, Farmingdale, NY, USA) and the oxidation-sensitive fluorescent probe dichloro-dihydro-fluorescein diacetate (DCFH-DA) to measure the intracellular ROS levels in the cells. We incubated cells (5.0 × 10^4^) at 37 °C in the dark for 60 min with DCFH-DA at a final concentration of 10 μM. Following three PBS washes, the cells were trypsinized and resuspended. We counted ROS-positive and DAPI-positive cells in four rectangular areas (0.75 mm 1.0 mm) on each slide for quantitative analysis and calculated the mean values. The percentage of ROS-positive cells was estimated using the formula (number of ROS-positive nuclei/number of DAPI-positive nuclei) × 100. This was expressed as the average of the four regions.

### 2.7. Statistical Analysis

We used the Steel–Dwass test to examine the data reported as means of standard deviation (SD). We used Fisher’s protected least significant difference test for post hoc analysis. We determined that a statistically significant difference existed at *p* < 0.05.

## 3. Results

### 3.1. Modification of AGE-Related Markers in RC-Derived Cells by Apocynin Treatment

**Figure 1:**
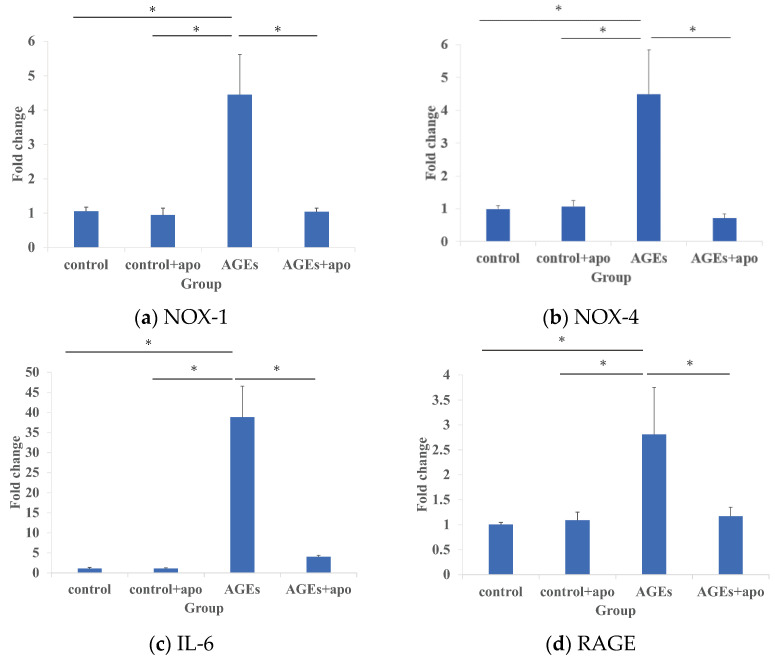
(**a**) Expression of NOX-1 mRNA in the AGEs group was significantly higher than in the other groups at 48 h. (**b**) Expression of NOX-4 mRNA; (**c**) expression of IL-6 mRNA; and (**d**) expression of RAGE mRNA showed similar findings to that of NOX-1. * *p* < 0.05.

The mRNA expression of NOX-1 in the AGEs group was significantly higher than that of the control and control apo+ groups (*p* < 0.05, *p* < 0.05, respectively) (Figure 1a). That of NOX-1 in the AGEs + apo group was significantly lower than that in the AGEs group (*p* < 0.05), though there was no difference between the control and control + apo groups (Figure 1a). The mRNA expressions of NOX-4, IL-6, and RAGE in the AGEs group were also significantly higher than those in the other groups (*p* < 0.05 for all) (Figure 1b–d).

### 3.2. Apocynin Resulted in Inhibition of Decreased RC-Derived Cell Viability with AGEs Stimulation

**Figure 2:**
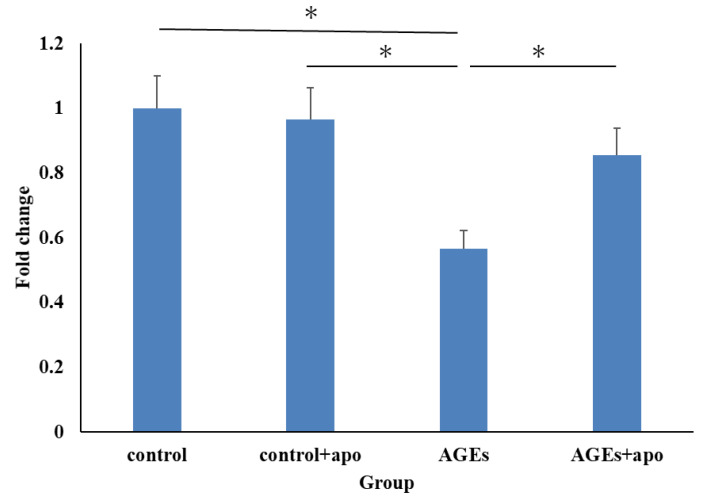
At 48 h, cell viability in the AGEs group was significantly lower than in the control + apo groups. That is, cell viability in the AGEs + apo group was significantly higher than in the AGEs group. However, there was no difference between the control, control + apo, and AGEs + apo groups. * *p* < 0.05.

At 48 h, cell viability in the AGEs group was significantly lower than that in the control and control + apo groups (*p* < 0.05, *p* < 0.05, respectively) (Figure 2). That in the AGEs + apo group was also lower than in the control and control + apo group, though there was no significant difference (Figure 2). Alternatively, that in the AGEs + apo group was significantly lower compared to the AGE+ group (*p* < 0.05) (Figure 2).

### 3.3. Cell Damage of AGEs to RC-Derived Cells and Their Inhibition Tendency with Apocynin

**Figure 3:**
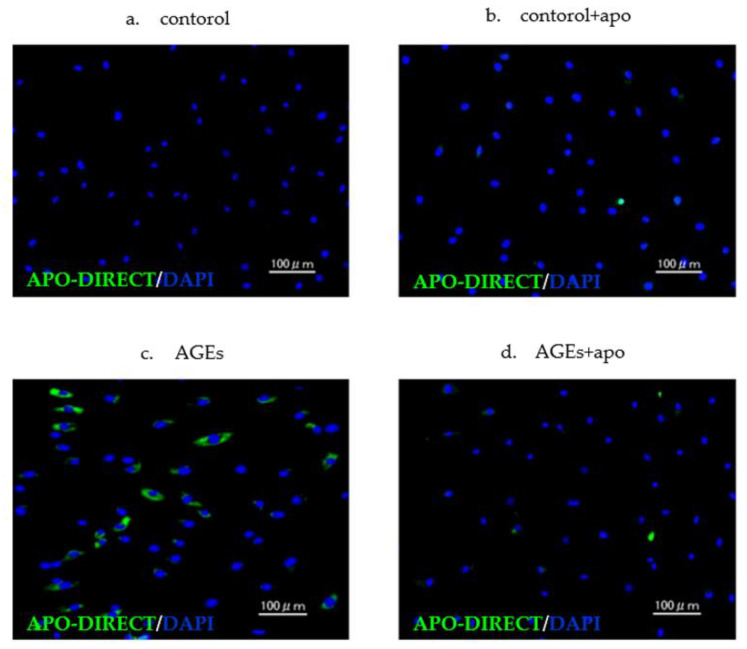
Immunofluorescence labeling depicting apoptotic (green) cells in each group. (**a**,**b**) There were few apoptotic cells in the control and control + apo groups. (**c**,**d**) There was apoptosis induction in AGEs and AGEs + apo groups. The amount of apoptotic cells in the AGEs + apo group was lower than that in the AGEs group.

**Figure 4:**
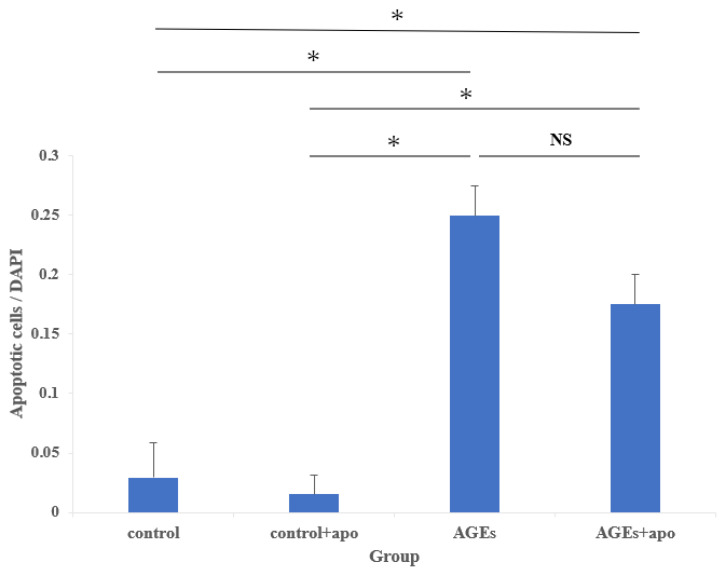
Quantification of the number of apoptotic cells. The number of apoptotic cells was analyzed by fluorescence intensity normalized to cell number. At 48 h, the number of apoptotic cells in the AGEs and AGEs + apo groups was considerably higher than in the control + apo group. In the AGEs + apo group, there were fewer apoptotic cells than in the AGEs group, though there was no significant difference between them. * *p* < 0.05. NS, no significance, DAPI, 4′,6-diamidino-2-phenylindole.

Apoptotic cells were seen in both AGEs and AGEs + apo groups. In contrast, there were few apoptotic cells in the control and control + apo groups (Figure 3). Abnormal nuclear morphology, such as nuclear fragmentation, was observed in apoptotic cells. A quantitative analysis of the apoptotic cells is shown in Figure 4. The amount of apoptotic cells in both the AGEs group and the AGEs + apo group was significantly higher than that in the control and control + apo groups, respectively (*p* < 0.05 for all) (Figure 4). The amount of cells in the AGE + apo group was lower relative to that of the AGEs group. Nevertheless, there was no significant difference (control, 0.03 ± 0.004; control + apo, 0.02 ± 0.002; AGEs, 0.25 ± 0.50; AGE + apo, 0.21 ± 0.30; Figure 4).

### 3.4. Downregulation of AGE-Related ROS Generation Was Observed in Apocynin-Treated RC Cells

**Figure 5:**
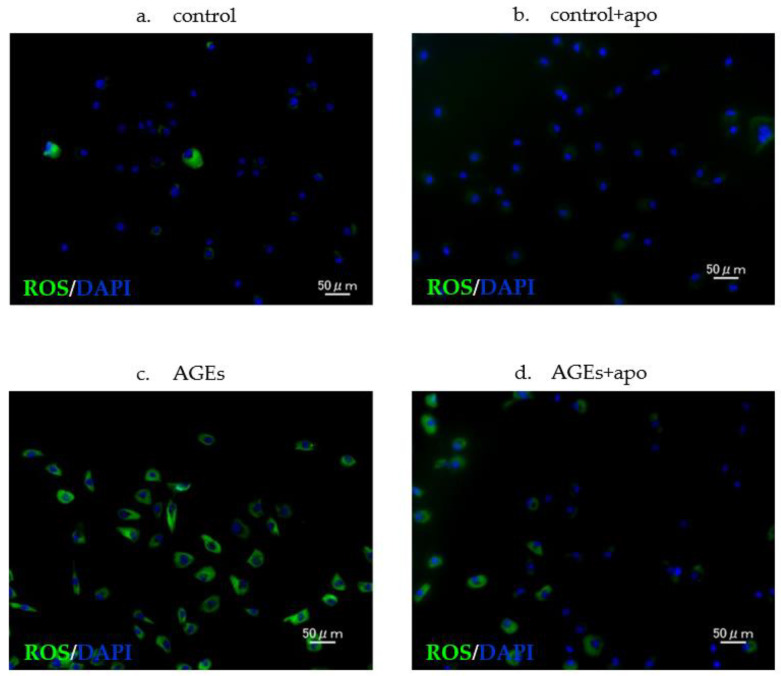
Fluorescence staining showing ROS. ROS production in rotator cuff-derived cells and nuclei (DAPI) (blue). (**a**,**b**) Low ROS production was observed in control + apo groups; (**c**,**d**) increased ROS production was observed in the AGEs group and the AGEs + apo group compared to the control and control + apo groups. ROS production was decreased in the AGEs + apo group compared to the AGEs group.

**Figure 6:**
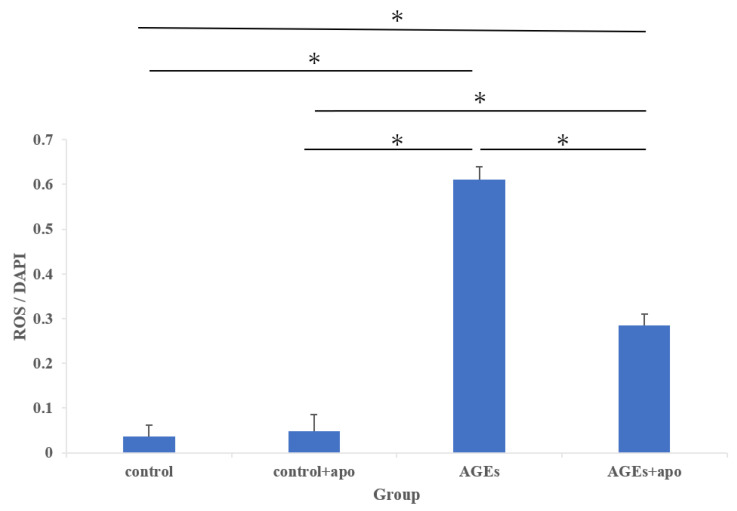
The quantification of ROS generation. The generation of reactive oxygen species was evaluated by fluorescence intensity normalized to cell number. ROS production in the AGEs group and the AGE + apo group was significantly higher than in control + apo groups at 48 h. ROS production in the AGEs + apo group was significantly lower than in the AGEs group. * *p* < 0.05. DAPI, 4′,6-diamidino-2-phenylindole.

Using DCFH-DA, intracellular ROS levels were detected. The cytoplasm of ROS-positive cells is stained green (Figure 5). The amount of ROS-positive cells in both the AGEs group and the AGE + apo group was significantly higher than that in control and control + apo groups, respectively (control, 0.20 ± 0.04; control + apo, 0.32 ± 0.06; AGEs, 0.61 ± 0.02; AGE + apo, 0.28 ± 0.02; *p* < 0.05 for all; Figure 6). The amount of cells in the AGE + apo group was significantly lower than that of the AGEs group (*p* < 0.05) (Figure 6). In the human RC cells, a significant reduction in AGE-related ROS generation was detected after apocynin treatment.

## 4. Discussion

The detrimental effects of AGE-related medical conditions are well explained in non-tendon tissue and are associated with several diabetic complications, such as cardiomyopathy, retinopathy, nephropathy, and endothelial dysfunction [4,5,6]. This study sought to address the effects of AGEs at the cellular level to better understand the factors that may contribute to the collapse and degeneration of tendon ECM under oxidative stress. Tendon and ligament damage remains an important issue in medicine and biomedical engineering and remains an unresolved issue. Unfortunately, in the case of musculoskeletal disorders such as tendon disorders, there are few studies that have provided detailed validation of animal models beyond structural and biomechanical similarities. Many intrinsic and extrinsic factors, such as age, weight, and physical exertion, influence the etiology of tendon disorders [24]. The factors contributing to tendon degeneration of diabetic tendons and the dismantling of collagen fibrils have not been widely characterized. Multiple vital mechanisms, including AGEs synthesis, activation of intracellular signaling molecules such as protein kinase C (PKC), and enhanced ROS production, appear to be involved in developing diabetic problems [25]. These data emphasize that hyperglycemia is unlikely to be the only cause of diabetic tendon lesions. To support this concept, we investigated the role of AGEs, which may interfere with regulating and maintaining tendon cell properties.

NOX is a significant cytosolic source of ROS production [26]. Previous research has found seven NOX isoforms [27]. Because they were the predominant isoforms in vascular smooth muscle cells, NOX-1 and NOX-4 were the principal ROS generators in the cardiovascular system [27]. According to Koike et al., AGEs dramatically boosted the expression of NOX-1 and NOX-4 mRNA in rat vascular smooth muscle cells [28]. According to Ueda et al., high glucose circumstances increased the expression of NOX-1 and IL-6 mRNA, as well as the production of ROS, though not when AGEs were stimulated [29]. Mifune et al. reported that the presence of AGEs increased the amount of ROS that accumulated in human rotator cuff-derived cells [19]. In this study, the AGEs group had greater levels of NOX-1 and NOX-4 mRNA expression and ROS accumulation, which is consistent with other studies showing that AGEs cause oxidative stress via activation of NOX [30].

Apocynin has been shown to effectively inhibit the increased NOX activity in diabetic aortas and restore nitric oxide synthase expression, thus breaking the vicious cycle that leads to diabetes-associated endothelial dysfunction [31]. In addition, Thallas-Bonke et al. reported that apocynin attenuated cytosolic superoxide and PKC activation by blockading NOX in diabetic nephropathy rats [32]. This study using human rotator cuff-derived cells also showed apocynin administration to reduce the expressions of NOX-1 and NOX-4 mRNA in the AGE + apo group relative to the AGE group. Furthermore, quantitative investigation of ROS accumulation revealed that apocynin therapy decreased intracellular ROS levels in response to AGEs stimulation.

RAGE is a pattern recognition inflammatory receptor that interacts with several ligands, including AGEs [32]. In the pathogenesis of diabetic nephropathy, the accumulation of AGEs and RAGE expressions play critical roles. In addition, RAGE activation by AGEs stimulates signal transmission and generates oxidative stress in endothelial cells via NOX activation [30]. In this study, RAGE mRNA expression was higher in the AGEs group than in any other group. These results might suggest AGEs–RAGE interaction, with AGEs alone producing ROS via NOX activation in the AGEs group. Furthermore, our results indicated that RAGE mRNA expression was significantly decreased by apocynin administration. This result might be attributed to the inhibition of NOX activation by apocynin because glycation and oxidation influence each [32].

According to several studies, AGEs cause oxidative stress, which damages DNA and causes cell death through a mitotic catastrophe. Approximately 20–30% of cells die after 24 to 36 h of incubation following AGE treatment, which frequently results in DNA damage and oxidative stress [33]. A recent paper reported that AGEs stimulation increased the apoptotic rate in rotator cuff-derived cells [19]. A growing amount of evidence suggests that excessive ROS production induces cellular damage and an apoptotic cascade through c-Jun N-terminal kinase (JNK) phosphorylation and Bax activation [16] and via caspase activation and control of the expression of Bcl-2 family proteins [34]. ROS enhance the overproduction process of apoptosis by raising caspase-3 activity and suppressing Bcl-2 expression, illustrating the interaction between oxidative stress and apoptosis [35]. Other studies have also shown that exogenous oxidative stress caused by H2O2 loading induced cellular apoptosis in human tendon fibroblasts [36]. Our results indicated that the amount of apoptotic cells was higher in the AGEs group, as with previous reports. Li et al. reported that in the testes of diabetic rats, apocynin significantly reduced ROS production and the amount of apoptotic cells [37]. In this study, the amount of apoptotic cells was decreased in the AGEs + apo group compared to the AGEs group, but there was no significant difference between the two groups. Nevertheless, apocynin suppressed apoptosis due to decreased ROS production in rotator cuff-derived cells.

In diabetic patients with flexor tenosynovitis, hyperplastic granulation tissue included a limited number of inflammatory cells [38]. Compared to the control group, the diabetic group’s Achilles tendons exhibited a higher density of type 1 collagen, increased VEGF expression, and increased immunostaining for nuclear factor kappa B (NFκB) p50 nuclear localization in the nucleus [39]. In addition, Ueda et al. reported that high glucose conditions upregulated mRNA expression for IL-6 [29]. Our study indicated that the expression of IL-6 mRNA was higher in the AGEs group and was decreased by apocynin administration. This result might be caused by apocynin, which inhibited NOX activation. AGEs–RAGE interaction promotes NOX activity, resulting in NFκB activation followed by the expression of inducible nitric oxide synthase and increased formation of free radicals [40].

Research into tendon injury utilizes many different animal models. However, the extent to which these species simulate clinical conditions and disease pathophysiology has not yet been critically evaluated. This study evaluates inflammation’s cellular and molecular features in human rotator cuff-derived cells. Previously, mouse and rat tendon cells were the closest to simulating human low proliferative capacity and inflammation with negligible effects. Nevertheless, rats and horses are the closest matches for human wound recovery kinetics [41]. Human tendon cell gene expression was similar to healthy mice, transiently inflammatory horses, and inflammatory sheep. Humans had the best concordance with mice and horses for tendon markers and collagen expression, horses for extracellular matrix remodeling genes, and rats for inflammatory mediators [41]. These findings imply that no one animal model adequately mimics human tendon cells and accurately reproduces the clinical condition. As a result of the multifactorial engagement of various critical pathways, we have shown that the cellular and molecular characteristics of inflammation in human tendon cells are related to the effects of AGE generation and its inhibitors. As no single animal model perfectly replicates the clinical condition and sufficiently emulates human tenocytes, this study showing the efficacy of apocynin on human rotator cuff cells is significant.

There are several limitations to this study. First, differences in age and variations in patients’ physiological and pathological status are the significant limitations of this study. The findings of this investigation were preliminary; consequently, human clinical application would necessitate additional research. Second, researchers should be cautious when extrapolating the results of this in vitro study to in vivo conditions. Other animal studies using aging or overuse models should be conducted to validate the effects of apocynin. Third, we did not assess ECM proteins susceptible to AGEs modification because of their slow turnover rate [42]. Under oxidative stress, there are multiple apoptosis-related pathways. However, the mediator of apoptotic signaling pathways has not been studied. Fourth, we assessed only the TUNEL assay. We employed tenocytes cultivated at passages 2 or 3, although phenotypic drift must be considered because the phenotype of tenocytes in culture rapidly shifts with each successive passage [43]. To better comprehend the mechanism, it may be necessary to experiment with an apocynin inhibitor. However, experiments with future in vivo apocynin inhibitors may be required to determine whether the risk of AGE-related degenerative tendon disorders in diabetics can be reduced.

## 5. Conclusions

Conclusively, AGE-induced oxidative stress upregulated the mRNA expression of NOX-1, NOX-4, IL-6, and RAGE. However, it also increased ROS production and the amount of apoptotic cells and deteriorated cell viability via NOX activation. In contrast, apocynin dramatically decreased ROS generation and cell death in rotator cuff-derived cells via inhibition of NOX and improved cell viability. Thus, apocynin could exert inhibitory effects for AGE-induced oxidative stress in rotator cuff-derived cells. Apocynin is a potential prodrug in preventing degenerative changes in the rotator cuff.

## Figures and Tables

**Table 1:** Patient background characteristics.

Characteristic	Patients Registered (n = 12)
Age (y), mean ± SD	64.8 ± 11.7 SD
Sex (%)	
Men	4 (33)
Women	8 (66)
Complications (%)	
Diabetes	2 (17)
Hypertension	3 (17)
Rotator cuff tear size (%) (Cofield classification)	Small: 6 (50); Medium: 5 (42); Large: 1 (17)
Fatty degeneration (%) (Goutallier classification)	Stage1: 5 (42); Stage2: 6 (50); Stage3: 1 (17)

**Table 2:** Primer sequences for quantitative real-time PCR analysis.

Gene	Oligonucleotide sequence
NOX-1	Forward 5′ GGTTTTACCGCTCCCAGCAGAA 3′
	Reverse 5′ CTTCCATGCTGAAGCCACGCTT 3′
NOX-4	Forward 5′ GCCAGAGTATCACTACCTCCAC 3′
	Reverse 5′ CTCGGAGGTAAGCCAAGAGTGT 3′
IL-6	Forward 5′ AGACAGCCACTCACCTCTTCAG 3′
	Reverse 5′ TTCTGCCAGTGCCTCTTTGCTG 3′
RAGE	Forward 5′ CACCTTCTCCTGTAGCTTCAGC 3′
	Reverse 5′ AGGAGCTACTGCTCCACCTTCT 3′
GAPDH	Forward 5′ GTCTCCTCTGACTTCAACAGCG 3′
	Reverse 5′ ACCACCCTGTTGCTGTAGCCAA 3′

NOX, nicotinamide adenine dinucleotide phosphate oxidase; IL-6, interleukin-6; RAGE, receptor for advanced glycation end-products; GAPDH, glyceraldehyde 3-phosphate dehydrogenase.

## Data Availability

All of the data are contained within the article.
